# A pivotal bridging study of lurbinectedin as second-line therapy in Chinese patients with small cell lung cancer

**DOI:** 10.1038/s41598-024-54223-5

**Published:** 2024-02-13

**Authors:** Ying Cheng, Chunjiao Wu, Lin Wu, Jun Zhao, Yanqiu Zhao, Lulu Chen, Ying Xin, Liang Zhang, Pinhua Pan, Xingya Li, Juan Li, Xiaorong Dong, Ke Tang, Emei Gao, Fei Yu

**Affiliations:** 1 Department of Oncology, Jilin Cancer Hospital, Changchun, 130000 China; 2 Department of Thoracic Oncology, Hunan Cancer Hospital, Changsha, 410013 China; 3 Department of Thoracic Oncology, Beijing Cancer Hospital, Beijing, 100142 China; 4 Department of Oncology, Henan Cancer Hospital, Zhengzhou, 450003 China; 5 Department of Respiratory Disease, Xiangya Hospital Central South University, Changsha, 410008 China; 6 Department of Oncology, The First Affiliated Hospital of Zhengzhou University, Zhengzhou, 450052 China; 7 Department of Oncology, Sichuan Cancer Hospital, Chengdu, 610041 China; 8 Center of Oncology, Union Hospital Tongji Medical College, Huazhong University of Science and Technology, Wuhan, 430022 China; 9 Clinical Research Center of Luye Pharma Group Ltd, Luye Life Sciences Group, Beijing, 100080 China; 10 School of Pharmacy, Yantai University, Yantai, 264005 China

**Keywords:** Cancer, Medical research, Oncology

## Abstract

This single-arm, multi-center clinical trial aimed to evaluate the safety, tolerability, DLT, recommended dose (RD), preliminary efficacy, and pharmacokinetics (PK) characteristics of lurbinectedin, a selective inhibitor of oncogenic transcription, in Chinese patients with advanced solid tumors, including relapsed SCLC. Patients with advanced solid tumors were recruited in the dose-escalation stage and received lurbinectedin in a 3 + 3 design (two cohorts: 2.5 mg/m^2^ and 3.2 mg/m^2^, IV, q3wk). The RD was expanded in the following dose-expansion stage, including relapsed SCLC patients after first-line platinum-based chemotherapy. The primary endpoints included safety profile, tolerability, DLT, RD, and preliminary efficacy profile, while the secondary endpoints included PK characteristics. In the dose-escalation stage, ten patients were included, while one patient had DLT in the 3.2 mg/m^2^ cohort, which was also the RD for the dose-expansion stage. At cutoff (May 31, 2022), 22 SCLC patients were treated in the ongoing dose-expansion stage, and the median follow-up was 8.1 months (range 3.0–11.7). The most common grade ≥ 3 treatment-related adverse events (TRAEs) included neutropenia (77.3%), leukopenia (63.6%), thrombocytopenia (40.9%), anemia (18.2%), and ALT increased (18.2%). The most common severe adverse events (SAEs) included neutropenia (27.3%), leukopenia (22.7%), thrombocytopenia (18.2%), and vomiting (9.1%). No treatment-related deaths occurred. The Independent Review Committee (IRC)-assessed ORR was 45.5% (95% CI 26.9–65.3). Lurbinectedin at the RD (3.2 mg/m^2^) showed manageable safety and acceptable tolerability in Chinese patients with advanced solid tumors, and demonstrates promising efficacy in Chinese patients with SCLC as second-line therapy.

**Trial registration:** This study was registered with ClinicalTrials.gov NCT04638491, 20/11/2020.

## Introduction

Being a highly aggressive subtype, small cell lung cancer (SCLC) accounts for approximately 15% of all lung cancers, with a poor 5-year survival rate of 7%^1,2^. At the time of diagnosis, the majority of SCLC patients exhibit extensive-stage disease^3^. Currently, platinum-based chemotherapy or a combination with immunotherapy remains the principal treatment regimen for these patients; however, rapid recurrence and resistance to treatment occur in nearly all the patients^4^. In the second-line therapy of SCLC, until lurbinectedin was approved by the Food and Drug Administration (FDA) in 2020^4^, topotecan was the only FDA-approved agent for patients with the platinum-sensitive disease who progressed ≥ 60 days after initiation of first-line chemotherapy since 1998. Nevertheless, topotecan usage gave unsatisfactory response rates as well as considerable hematological and gastrointestinal toxicities in that population. Notably, the recommended dose (RD) of topotecan in Chinese patients with relapsed SCLC is lower than that in Caucasian patients (1.25 mg/m^2^ vs. 1.5 mg/m^2^), suggesting the interplay of ethnic differences in the acceptability of foreign clinical data.

Lurbinectedin, developed by Pharma Mar, is a selective inhibitor of oncogenic transcription and is used for treating solid tumors. Lurbinectedin works by covalently binding to the central guanine of various nucleotide triplets in the DNA minor groove and forming adducts that induce double-strand breaks and disrupt DNA–protein interactions and RNA transcription, thereby resulting in DNA damage and apoptosis^5,6^. Other antitumor mechanisms of lurbinectedin include inducing immunogenic cell death (ICD), stimulating anticancer immunity^7^, and reducing tumor-associated macrophages (TAMs) in the tumor microenvironment^8^, etc. Several in vitro and in vivo studies have reported antitumor activities of lurbinectedin in a variety of human solid tumors, such as lung cancer, colon cancer, ovarian cancer, gastric cancer, etc.^9,10^. In June 2020, the FDA conditionally approved lurbinectedin (3.2 mg/m^2^, 1 h IV, q3wk) for treating adult patients with metastatic SCLC with disease progression on or after platinum-based chemotherapy^11^. Lurbinectedin was also included in the National Comprehensive Cancer Network (NCCN) guidelines of SCLC, from July 2020^12^. Furthermore, lurbinectedin was the first FDA-approved drug in over 20 years in the second line therapy for patients with metastatic SCLC^4^.

The FDA’s accelerated approval for lurbinectedin was granted based on the SCLC cohort results in a single-arm, open-label, phase II basket trial (PM1183-B-005–14, NCT02454972), in which 105 SCLC patients (including 1 Asian) who had been pre-treated with one previous platinum-based chemotherapy-containing line of treatment were included and treated with lurbinectedin (3.2 mg/m^2^, 1 h IV, q3wk) as the second-line therapy until disease progression or unacceptable toxicity. The phase II basket trial results showed that, at data cutoff, after a median follow-up of 17.1 months, the overall response rate (ORR) assessed by the investigator was 35.2% (95% CI 26.2–45.2), with a median duration of response (mDOR) of 5.3 (95% CI 4.1–6.4) months; the median progression-free survival (mPFS) was 3.5 (95% CI 2.6–4.3) months, and the median overall survival (mOS) was 9.3 (95% CI 6.3–11.8) months. The most common toxicities included myelosuppression and hepatotoxicity^13^.

Lurbinectedin was also investigated in combination with doxorubicin in a phase III study (ATLNATIS trial, NCT02566993), before the start of the basket trial, on the basis of preclinical evidence of potential synergistic effects as well as the preliminary results from a phase I clinical study. ATLANTIS was a multicenter, randomized trial which evaluated lurbinectedin 2.0 mg/m^2^ in combination with doxorubicin 40.0 mg/m^2^ every 21 days versus physician’s choice of either intravenous topotecan or cyclophosphamide/doxorubicin/vincristine (CAV) with mandatory G-CSF prophylaxis in both groups in adult patients with SCLC who relapsed after one previous platinum-containing chemotherapy regimen. However, the combination of lurbinectedin and doxorubicin did not meet the primary endpoint of overall survival and did not show a statistical survival advantage versus control in patients with relapsed SCLC, although lurbinectedin plus doxorubicin showed a favourable haematological safety profile compared with control. A phase III confirmatory study (LAGOON trial, NCT05153239) comparing the FDA-approved dose (3.2 mg/m^2^, q3wk) of lurbinectedin either as monotherapy or in combination with irinotecan versus investigator’s choice of irinotecan or topotecan is currently ongoing^14^.

Lurbinectedin has not been approved in China, and its character in the Chinese population has not been evaluated yet. To investigate the safety, tolerability, dose-limiting toxicity (DLT), recommended dose (RD), preliminary efficacy, and pharmacokinetics (PK) characteristics of lurbinectedin on Chinese advanced solid tumor patients, including relapsed SCLC, we conducted this study (LY01017/CT-CNH-101, NCT04638491, 20/11/2020) in China, to bridge the results of phase II basket trial.

## Methods

### Study design, participants, and treatments

This is a single-arm, open-label, multicenter, bridging study, including dose-escalation and dose-expansion stages. The main eligibility criteria are described in the Supplementary Appendix. In the dose-escalation stage, the patients recruited by the study investigator in Jilin Cancer Hospital received lurbinectedin in a classical 3 + 3 design. Two dose levels (DLs): DL1 (2.5 mg/m^2^) and DL2 (3.2 mg/m^2^), were evaluated as both were administered as a 1-h intravenous infusion once every 3 weeks; no dose escalation beyond DL2 was allowed. The RD was defined as the highest dose at which < 1/3 patients displayed dose-limiting toxicities (DLTs, which are detailed below) in Cycle 1. Primary granulocyte colony-stimulating factor (G-CSF) prophylaxis was not allowed during Cycle 1. If > 1 patient had neutropenia-associated DLTs in Cycle 1 at DL2, then this dose level would be used as the starting dose for a second dose escalation with primary G-CSF prophylaxis during Cycle 1. After defining RD, the study entered the dose-expansion stage, and patients were recruited by the study investigators in eight hospitals in the Chinese mainland and treated with single-agent lurbinectedin at the RD defined in the dose-escalation stage (with or without G-CSF support).

In the dose-escalation stage, DLTs were defined as any adverse events (AEs) or laboratory examination abnormalities related to lurbinectedin that occurred in the first cycle and followed the required criteria (graded per NCI-CTCAE 5.0): grade 4 neutropenia for ≥ 3 days; ≥ grade 3 febrile neutropenia; ≥ grade 3 neutropenia combined with sepsis or other serious infections; grade 4 thrombocytopenia, or grade 3 thrombocytopenia complicated by obvious hemorrhage or requiring platelet transfusion; grade 4 anemia; other grades 3/4 none-hematological AEs suspected to be related with lurbinectedin except nausea/vomiting (unless no obvious remission after treatment for 2 weeks), grade 3 diarrhea for < 24 h or could be obviously relieved within 2 weeks through medical intervention, grade 3 weakness for < 5 days, allergic reactions, hair loss, and simple biochemical abnormalities unrelated with the clinical disease.

The study protocol was approved by an independent local ethics committee of each participating hospital. The study was done in accordance with the *Declaration of Helsinki*, the Good Clinical Practice international guidelines, and the local regulations for clinical trials. Signed informed consent was obtained from all patients before any study-specific procedure started.

### Procedures

In the dose-escalation stage, patients were given lurbinectedin from 2.5 to 3.2 mg/m^2^, based on the a 3 + 3 design rule. Finally, a dose of 3.2 mg/m^2^ without G-CSF support was defined as the RD for the dose-expansion stage.

In the dose-expansion stage, all patients were given a starting dose of 3.2 mg/m^2^ lurbinectedin and antiemetic prophylaxis. Treatment delays and dose reductions were permitted for managing toxic effects at the investigator’s discretion. However, patients requiring > 2 dose reductions (from 3.2 to 2.6 mg/m^2^ and then to 2.0 mg/m^2^) were to be withdrawn form the trial.

**Figure 1:**
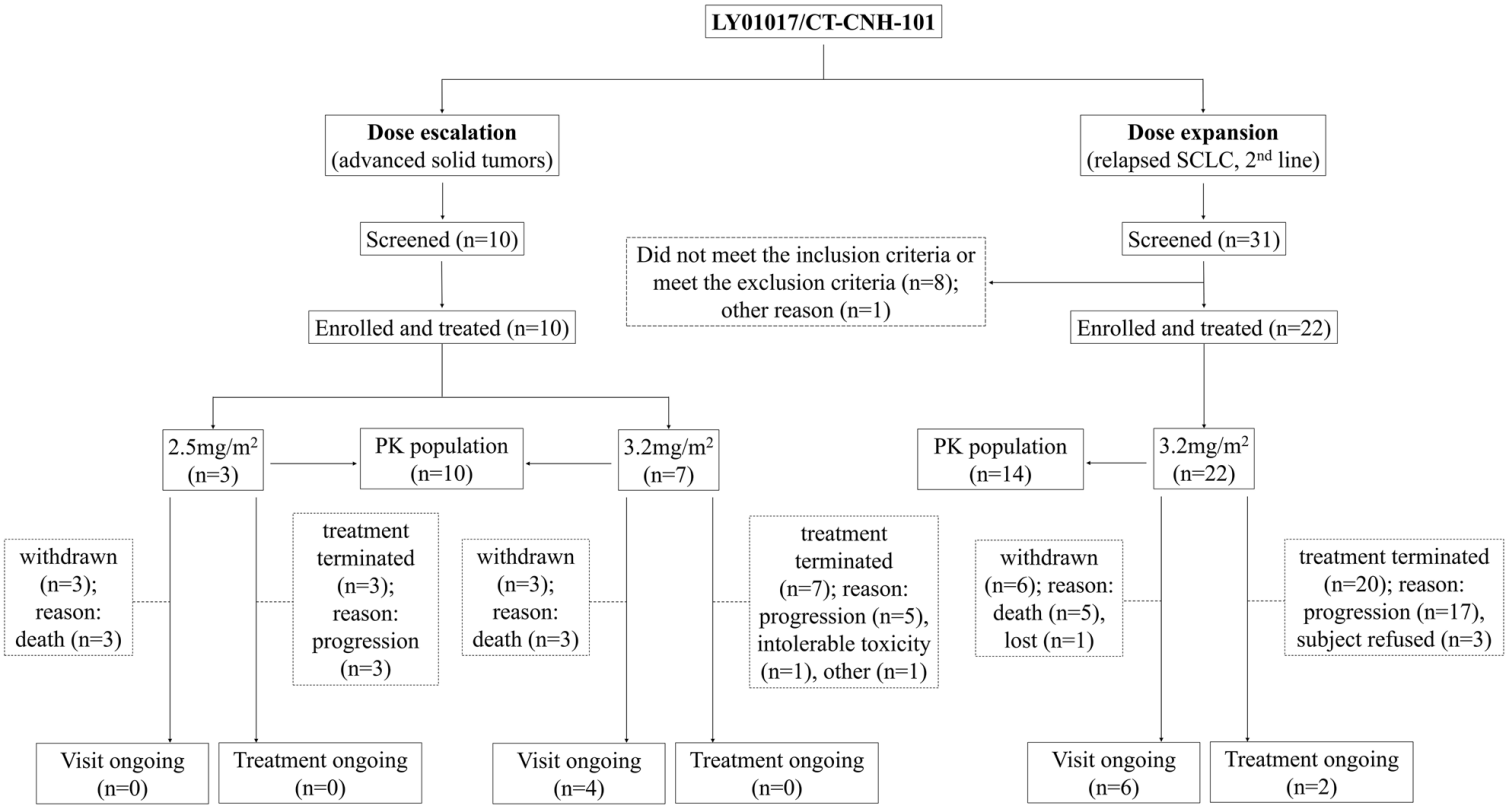
Study design and subject flow. *PK* pharmacokinetics.

For both stages, all the patients received lurbinectedin until disease progression [defined by the Response Evaluation Criteria in Solid Tumors (RECIST) criteria] or unacceptable toxicity (as per the investigator’s decision), except for excluded patients. The antitumor activities were evaluated using a radiological assessment [contrast-enhanced computed tomography (CT) or magnetic resonance imaging (MRI) scan] in accordance with RECIST1.1, once every 6 weeks (± 7 days) after the first dose. The tumors were also evaluated by the independent review committee (IRC) through a masked review of the radiological results through de-identified images; the statistical analysis of ORR was mainly based on the IRC results (Fig. 1).

### Pharmacokinetic profile evaluation

The PK sampling was conducted on 3 and 21 subjects in the 2.5 mg/m^2^ and 3.2 mg/m^2^ groups during the trial, respectively. For each subject, 16 and 9 blood samples were collected in the 1st and the 2nd cycles for PK analysis, respectively.

### Endpoints

The primary observation endpoints included safety profile, tolerability, DLT, and RD of lurbinectedin in Chinese advanced solid tumor patients, as well as the preliminary efficacy of lurbinectedin at RD as the second-line therapy in Chinese SCLC patients, while the secondary endpoints included pharmacokinetic parameters.

### Statistical analysis

The overall objective response, overall survival, and PFS were assessed in the intention-to-treat population, comprising all enrolled participants. The response duration was assessed in all the participants who had a confirmed complete or partial response (PR). Moreover, the safety profile was assessed in the as-treated population, defined as all participants who took at least one study treatment dose.

We used SAS (version 9.4) for all statistical analyses. We used the binomial method (Wilson binomial) to assess the 95% confidence interval of the overall objective response rate (ORR). We also estimated the overall survival, PFS, and response duration using the Kaplan–Meier method.

Based on a sample size of 22 subjects, if the observed ORR was 30.5%, our study would have an 85% power to ensure that the point estimate of ORR is greater than the lower bound of 95% CI for ORR from the PM1183-B-005-14 SCLC cohort study, which is 21.9%. The uncertainty of the ORR estimate was calculated by a 95% exact binomial confidence interval.

### Ethics approval and consent to participate

The study was approved by the ethics committee of Jilin Cancer Hospital (202008-044-01). All patients provided written informed consent.

## Results

Between November 24, 2020, and May 31, 2022, a total of 32 patients were enrolled; all were treated with lurbinectedin and included for the evaluation of safety analysis. Ten patients with various advanced solid tumors (4 breast cancer, 2 SCLC, 2 sarcomas, 1 rectal cancer, and 1 pancreatic cancer) were included in the dose-escalation stage, while 22 relapsed SCLC patients after first-line platinum-based chemotherapy were enrolled in the dose-expansion stage, respectively. One baseline CNS metastasis patient was included in the dose-expansion stage because of the missed diagnosis of a minor metastatic lesion in the MRI scan of the brain during the screening. The investigators judged this patient as a potential beneficiary of lurbinectedin and thus continued the treatment, albeit this case was considered a protocol deviation. Among the 22 patients, 17 (77.3%) were males, and 20 (90.9%) of them were diagnosed at the extensive stage; the median age was 58; the chemotherapy-free interval (CTFI) was < 90 days in 8 (36.4%) patients and > 90 days in 14 (63.6%) patients. There were 14/22 (63.6%) patients who previously received immunotherapy before their dose-expansion stage enrollment (Tables 1, 2).

**Table 1:** Baseline characteristics in the dose-escalation stage.

	2.5 mg/m^2^ (n = 3)	3.2 mg/m^2^ (n = 7)	Total (n = 10)
Sex			
Male	1 (33.3%)	3 (42.9%)	4 (40.0%)
Female	2 (66.7%)	4 (57.1%)	6 (60.0%)
Median age, years	38 (37–60)	57 (33–71)	55.5 (33–71)
≥ 65	0	1 (14.3%)	1 (10.0%)
Median BMI (kg/m^2^)	26.7 (19.5–27.3)	24.2 (18.1–37.3)	25.3 (18.1–37.3)
ECOG performance status			
0	0	1 (14.3%)	1 (10.0%)
1	3 (100%)	6 (85.7%)	9 (90.0%)
2	0	0	0
Median number of tumour sites at baseline	2 (2–11)	2 (1–5)	2 (1–11)
≥ 3 sites	1 (33.3%)	2 (28.6%)	3 (30.0%)
Bulky disease (one lesion > 50 mm, IRC-assessed)	1 (33.3%)	3 (42.9%)	4 (40.0%)
CNS involvement	0	0	0
Median number of previous therapy lines	2 (1–3)	1 (1–3)	1 (1–3)
Type of tumors			
SCLC	1 (33.3%)	1 (14.3%)	2 (20.0%)
Others	2 (66.7%)	6 (85.7%)	8 (80.0%)
Previous immunotherapy	1 (33.3%)	0	1 (10.0%)

*BMI* body mass index, *ECOG* Eastern Cooperative Oncology Group, *IRC* Independent Review Committee, *CNS* central nervous system, *SCLC* Small Cell Lung Cancer.

**Table 2:** Baseline characteristics in the dose-expansion stage.

	All treated patients (n = 22)
Sex	
Male	17 (77.3%)
Female	5 (22.7%)
Median age, years	58 (52–69)
≥ 65	6 (27.3%)
≥ 75	0
Median BMI (kg/m^2^)	24.7 (20.7–30.1)
ECOG performance status	
0	6 (27.3%)
1	16 (72.7%)
2	0
Disease stage at diagnosis	
Limited	2 (9.1%)
Extensive	20 (90.9%)
Median number of tumour sites at baseline	1 (0–5)
≥ 3 sites	3 (13.6%)
Bulky disease (one lesion > 50 mm, IRC-assessed)	8 (36.4%)
CNS involvement	1 (4.5%)
Median number of previous therapy lines	1 (1–1)
Previous immunotherapy	14 (63.6%)
Chemotherapy-free interval (CTFI)	3.4 (0.7–34.9)
≥ 90 days	14 (63.6%)
< 90 days	8 (36.4%)
Best response to previous platinum therapy	
Complete response	0
Partial response	11 (50.0%)
Stable disease	6 (27.3%)
Disease progression	0
Unknown	5 (22.7%)

*BMI* body mass index, *ECOG* Eastern Cooperative Oncology Group, *IRC* Independent Review Committee, *CNS* central nervous system, *CTFI* chemotherapy-free interval.

At cutoff, the treatment cycles comprising total and median cycles were 43, 3 (range 1–14) and 132, 6 (range 1–12) in the dose-escalation and dose-expansion stages, respectively. The median total exposure dosage was 13.3 mg (range 5–89.6 mg) and 28.4 mg (range 5.4–74.5 mg) in the dose-escalation and dose-expansion stages, respectively. In the dose-escalation stage, no patients received ≥ 6 cycles in the 2.5 mg/m^2^ cohort, but 3/7 (42.9%) patients received ≥ 6 cycles in the 3.2 mg/m^2^ cohort. There were 12/22 (54.5%) patients who received ≥ 6 cycles in the dose-expansion stage. No dose reduction occurred in the dose-escalation stage, while the doses were reduced in 7 (31.8%) patients and 8 (6.1%) cycles in the dose-expansion stage because of treatment-related AEs. Similarly, treatment-related dose delay occurred in 2 (20.0%) patients and 2 (4.7%) cycles, as well as 8 (36.4%) patients, and 12 (9.1%) cycles in the dose-escalation and dose-expansion stages, respectively. The most common causes for both the dose reduction and delay were hematological toxicities (neutropenia, leukopenia, and thrombocytopenia). Moreover, two patients in the 3.2 mg/m^2^ cohort in the dose-escalation stage discontinued treatment because of treatment-emergent adverse events (TEAEs) (gastrointestinal disorders and hepatotoxicity), while only 1 (4.5%) patient in the dose-expansion stage discontinued the treatment due to TEAEs (grade 4 ALT increased and grade 3 AST increased).

With regard to safety and tolerability, in the dose-escalation stage, no DLT was observed in the first 3 patients who undertook 2.5 mg/m^2^ lurbinectedin. In the 3.2 mg/m^2^ groups, only 1 DLT (grade 4 neutropenia lasting ≥ 3 days) was observed. Additionally, 1 patient was considered not evaluable for DLT observation because of being given medical intervention for grade 3 ALT/AST increased during the DLT observation period. In the dose-escalation stage, 10 (100%) patients displayed one TEAE regardless of the relationship, and 9 (90.0%) patients had grades ≥ 3 TEAEs. The most common grade ≥ 3 TEAEs (> 10% patients) were hematological disorders like neutropenia (7, 70.0%), lymphopenia (5, 50.0%), leukopenia (5, 50.0%), and thrombocytopenia (2, 20.0%); all TEAEs were treatment-related occurrences. Severe adverse events (SAEs) occurred in two patients, including a grade 3 atrial fibrillation (2.5 mg/m^2^ cohort) and a grade 3 ALT/AST increased (3.2 mg/m^2^ cohort), which were treatment-related and required prolonged hospitalization. In the dose-expansion stage, 22 (100%) patients showed at least one TEAE regardless of the relationship, and 19 (86.4%) patients had grades ≥ 3 TEAEs. The most common grade ≥ 3 TEAEs (> 5% patients) were mostly hematological and liver disorders, including neutropenia (17, 77.3%), leukopenia (14, 63.6%), thrombocytopenia (9, 40.9%), anemia (4, 18.2%), ALT increased (4, 18.2%), AST increased (2, 9.1%), vomiting (2, 9.1%), and infectious pneumonia (2, 9.1%). All these TEAEs except one of infectious pneumonia were treatment-related occurrences. Furthermore, SAEs were reported in 11 (50.0%) patients and were regarded as treatment-related except a grade 4 ketoacidosis and grade 3 infectious pneumonia. The most common SAEs (> 5% patients) included neutropenia (6, 27.3%), leukopenia (5, 22.7%), thrombocytopenia (4, 18.2%), and vomiting (2, 9.1%) (Table 3, Table S1). At the data cutoff, no cases of sepsis and Hy’s law were reported. No treatment-related deaths occurred, while 5 (50.0%) and 4 (18.2%) patients in the dose-escalation and dose-expansion stages died from disease progression, respectively; two other patients died due to non-progression reasons.

**Table 3:** TEAEs worst grade ≥ 3 per patient in dose-escalation stage and dose-expansion stage.

MedDRA SOC/PT	Dose-escalation		Dose-expansion
	2.5 mg/m^2^	3.2 mg/m^2^	3.2 mg/m^2^
	n (%)	n (%)	n (%)
Patients with any grade ≥ 3 TEAEs	2 (66.7%)	7 (100%)	19 (86.4%)
Investigations	2 (66.7%)	7 (100%)	19 (86.4%)
Neutrophil count decreased	1 (33.3%)	6 (85.7%)	17 (77.3%)
White blood cell count decreased	0	5 (71.4%)	14 (63.6%)
Platelet count decreased	0	2 (28.6%)	9 (40.9%)
Alanine aminotransferase increased	0	1 (14.3%)	4 (18.2%)
Aspartate aminotransferase increased	0	1 (14.3%)	2 (9.1%)
Gamma-glutamyltransferase increased	0	0	1 (4.5%)
Lymphocyte count decreased	0	0	1 (4.5%)
Prolonged QT interval	1 (33.3%)	0	0
Blood and lymphatic system disorders	0	0	5 (22.7%)
Anemia	0	0	4 (18.2%)
Febrile neutropenia	0	0	1 (4.5%)
Gastrointestinal disorders	0	0	3 (13.6%)
Vomiting	0	0	2 (9.1%)
Nausea	0	0	1 (4.5%)
General disorders and administration site conditions	0	0	2 (9.1%)
Fatigue	0	1 (14.3%)	1 (4.5%)
Edema peripheral	0	0	1 (4.5%)
Metabolism and nutrition disorders	0	0	1 (4.5%)
Hypermagnesemia	0	0	1 (4.5%)
Hepatobiliary disorders	0	0	1 (4.5%)
Hepatic function abnormal	0	0	1 (4.5%)
Cardiac disorders	0	0	0
Fibrillation	1 (33.3%)	0	0
Infections and infestations	0	0	1 (4.5%)
Pneumonia	0	0	1 (4.5%)

*TEAE* treatment-emergent adverse events, *MedDRA* medical dictionary for regulatory activities (v.25.0), *SOC* system organ class, *PT* preferred term.

In the dose-escalation stage, the median follow-up was 4.6 months (range 4.0–12.6) and 13.2 months (range 1.5–14.9) in the 2.5 mg/m^2^ cohort and 3.2 mg/m^2^ cohort, respectively. A PR was observed in one soft tissue sarcoma patient in the 3.2 mg/m^2^ cohort; thus, ORR was 14.3% (95% CI 2.6–51.3) at this dose level, assessed by IRC or investigators. Notably, another sarcoma patient (renal leiomyosarcoma) benefited from 3.2 mg/m^2^ lurbinectedin with long-term stable disease (SD, in 6 assessments) until the disease progressed after treatment discontinuation due to the COVID-19 pandemic. In the 3.2 mg/m^2^ cohort, IRC-assessed DCR (i.e., CR + PR + SD) was 100% (95% CI 64.6–100.0) (Table 4, Supplementary Figs. S1, S2). In the dose-expansion stage, the median follow-up was 8.1 months (range 3.0–11.7). IRC-assessed ORR (the primary endpoint of efficacy evaluation) was 45.5% (95% CI 26.9–65.3) (10 PRs), which was coincident with the investigator’s results. The IRC-assessed mDOR, DCR, and mPFS were 4.2 months (95% CI 2.7–inf), 90.9% (95% CI 72.2–97.5), and 5.6 months (95% CI 4.1–6.9), respectively. According to the investigator’s assessment, mDOR, DCR, and mPFS were 2.9 months (95% CI 2.8–inf), 81.8% (95% CI 61.5–92.7), and 4.2 months (95% CI 4.0–5.4), respectively. With 17 cases alive being censored, the mOS was 11.0 months (95% CI 9.2–inf) in the overall population (Table 5, Supplementary Figs. S3–S7).

**Table 4:** Overall efficacy of lurbinectedin treatment assessed by IRC and the investigator in the dose-escalation stage.

Assessment	2.5 mg/m^2^ (n = 3)		3.2 mg/m^2^ (n = 7)		All patients (n = 10)	
	IRC	Investigator	IRC	Investigator	IRC	Investigator
RECIST responses						
Complete response	0	0	0	0	0	0
Partial response	0	0	1 (14.3%)	1 (14.3%)	1 (10.0%)	1 (10.0%)
Stable disease	1 (33.3%)	1 (33.3%)	6 (85.7%)	5 (71.4%)	7 (70.0%)	6 (60.0%)
Progressive disease	2 (66.7%)	2 (66.7%)	0	1 (14.3%)	2 (20.0%)	3 (30.0%)
Not evaluable	0	0	0	0	0	0
Overall response, % (95% CI)	0% (0–56.2)	0% (0–56.2)	14.3% (2.6- 51.3)	14.3% (2.6–51.3)	10.0% (1.8–40.4)	10.0% (1.8–40.4)
Disease control, % (95% CI)	33.3% (6.2–79.2)	33.3% (6.2–79.2)	100.0% (64.6–100.0)	85.7% (48.7–97.4)	80.0% (49.0–94.3)	70.0% (39.7–89.2)
Duration of response						
Disease progression, relapse, or death events in responding patients, n/N (%)	0	0	0	0	0	0
Censored*, n/N (%)	0	0	1/1(100.0%)	1/1(100.0%)	1/1(100.0%)	1/1(100.0%)
Median duration of response, months (95% CI)	NR (inf–inf)	NR (inf–inf)	NR (inf–inf)	NR (inf–inf)	NR (inf–inf)	NR (inf–inf)
Progression-free survival						
Progression-free survival events, n (%)	3 (100%)	3 (100%)	1 (14.3%)	4 (57.1%)	4 (40.0%)	7 (70.0%)
Censored*, n (%)	0	0	6 (85.7%)	3 (42.9%)	6 (60.0%)	3 (30.0%)
Median progression-free survival, months (95% CI)	1.6 (1.4–inf)	1.6 (1.4–inf)	12.2 (inf–inf)	4.2 (1.3–inf)	12.2 (1.4–inf)	2.7 (1.3–inf)
Overall survival						
Deaths	3 (100%)		3 (42.9%)		6 (60.0%)	
Censored*	0		4 (57.1%)		4 (40.0%)	
Median overall survival, months (95% CI)	4.4 (3.8–inf)		13.3 (1.4–inf)		12.4 (1.4–inf)	

*RECIST* response evaluation criteria in solid tumors, *IRC* Independent Review Committee.

*Patients censored due to disease not progressed or still being alive.

**Table 5:** Efficacy assessed by IRC and the investigator in the dose-expansion stage.

Assessment	All patients (n = 22)		CTFI < 90 days (n = 8)		CTFI ≥ 90 days (n = 14)	
	IRC	Investigator	IRC	Investigator	IRC	Investigator
RECIST responses						
Complete response	0	0	0	0	0	0
Partial response	10 (45.5%)	10 (45.5%)	3 (37.5%)	3 (37.5%)	7 (50.0%)	7 (50.0%)
Stable disease	10 (45.5%)	8 (36.4%)	5 (62.5%)	3 (37.5%)	5 (35.7%)	5 (35.7%)
Progressive disease	1 (4.5%)	3 (13.6%)	0	2 (25.0%)	1 (7.1%)	1 (7.1%)
Not evaluable	1 (4.5%)	1 (4.5%)	0	0	1 (7.1%)	1 (7.1%)
Overall response, % (95% CI)	45.5% (26.9–65.3)	45.5% (26.9–65.3)	37.5% (13.7–69.4)	37.5% (13.7–69.4)	50.0% (26.8–73.2)	50.0% (26.8–73.2)
Disease control, % (95% CI)	90.9% (72.2–97.5)	81.8% (61.5–92.7)	100.0% (67.6–100.0)	75.0% (40.9–92.9)	85.7% (60.1–96.0)	85.7% (60.1–96.0)
Duration of response						
Disease progression, relapse, or death events in responding patients, n/N (%)	4/10 (40.0%)	7/10 (70.0%)	1/3 (33.3%)	1/3 (33.3%)	3/7 (42.9%)	6/7 (85.7%)
Censored*, n/N (%)	6/10 (60.0%)	3/10 (30.0%)	2/3 (66.7%)	2/3 (66.7%)	4/7 (57.1%)	1/7 (14.3%)
Median duration of response, months (95% CI)	4.2 (2.7–inf)	2.9 (2.8–inf)	NR (4.0, –)	NR (2.9–inf)	4.2 (2.7–inf)	2.9 (2.8–inf)
Progression-free survival						
Progression-free survival events, n (%)	11 (50.0%)	17 (77.3%)	4 (50.0%)	5 (62.5%)	7 (50.0%)	12 (85.7%)
Censored*, n (%)	11 (50.0%)	5 (22.7%)	4 (50.0%)	3 (37.5%)	7 (50.0%)	2 (14.3%)
Median progression-free survival, months (95% CI)	5.6 (4.1–6.9)	4.2 (4.0–5.4)	6.6 (3.0–inf)	4.4 (0.5–inf)	5.4 (4.1–inf)	4.2 (4.0–5.4)
Overall survival						
Deaths	5 (22.7%)		3 (37.5%)		2 (14.3%)	
Censored*	17 (77.3%)		5 (62.5%)		12 (85.7%)	
Median overall survival, months (95% CI)	11.0 (9.2–inf)		9.2 (3.0–inf)		11.0 (inf–inf)	

*RECIST* response evaluation criteria in solid tumors, *IRC* Independent Review Committee, *CTFI* chemotherapy-free interval; one patient was not evaluable because of withdrawal.

*Patients censored due to disease not progressed or still being alive.

The efficacy of lurbinectedin in the dose-expansion stage was also evaluated by stratifying the 22 patients with CTFI. In patients with CTFI ≥ 90 days, IRC-assessed and investigator-assessed ORR had the same values: 50.0% (95% CI 26.8–73.2), and so were the DCR values: 85.7% (95% CI 60.1–96.0). The IRC- and investigator-assessed mDOR and mPFS values were 4.2 months (95% CI 2.7–inf) and 5.4 months (95% CI 4.1–inf), as well as 2.9 months (95% CI 2.8–inf) and 4.2 months (95% CI 4.0–5.4), respectively; the mOS was 11.0 months (inf–inf) with 12 (85.7%) censored alive cases. By comparison, in patients with CTFI < 90 days, IRC- and investigator-assessed ORR were 37.5% (95% CI 13.7–69.4), while the IRC-assessed DCR, mDOR, and mPFS were 100.0% (95% CI 67.6–100.0), NR, and 6.6 months (95% CI 3.0–inf), and the investigator-assessed results were 75.0% (95% CI 40.9–92.9), NR, and 4.4 months (95% CI 0.5–inf), respectively; the mOS was 9.2 months (3.0–inf) with 5 (62.5%) censored alive cases (Table 5).

In PK analysis, the drug concentration peak in plasma was observed immediately at the end of infusion, and the concentration decreased rapidly after the infusion. The plasma concentration after the 3.2 mg/m^2^ dosage was higher than the 2.5 mg/m^2^ dose. Before the Cycle 2 administration, the plasma concentration of all the patients in the two dose groups was below the Lower Limit of Quantification (Supplementary Figs. S8, S9), while a comparison of the C_max_ of the 2 cycles yielded no obvious accumulation. Additionally, the AUC_0–∞_ in Cycle 2 was larger than that in Cycle 1, because of fewer blood collection points in Cycle 2 which could overestimate the AUC_0–∞_ in Cycle 2 (Table S2).

## Discussion

Our study was the first clinical study conducted on lurbinectedin in Chinese patients, as well as the first dose-expansion study with an FDA-approved dosage (3.2 mg/m^2^, 1 h IV q3wk, without G-CSF primary prophylaxis) in Asian SCLC patients as second-line therapy.

The safety profile analysis in the dose-escalation stage suggested that hematological toxicities (e.g., neutropenia, leucopenia, and thrombocytopenia) were the main adverse reactions; however, they were manageable and tolerable as well as coincident with the findings from other studies. Notably, a case of grade 3 atrial fibrillation was reported as an SAE in the 2.5 mg/m^2^ group. However, based on a previous study (NCT02451007), significant effects of lurbinectedin administration on the QT interval of patients with solid malignancies at a dose of 3.2 mg/m^2^ q3wk have been ruled out^15^. The lurbinectedin’s safety profiles observed in our dose-expansion stage were similar to the basket trial results of the SCLC cohort, although with higher incidences of hematological and liver disorders. The different incidences may be attributed to the ethnic differences between the two studies and could be explained by the characteristic antitumor and metabolic activities of lurbinectedin in the body. Besides, according to the Investigator’s Brochure of Lurbinectedin (Aug, 2023), elevated creatine phosphokinase (CPK) and muscular adverse events have been reported in lurbinectedin historical studies. Specifically, CPK increases were reported in 9.2% of patients treated with single-agent lurbinectedin at 3.2 mg/m^2^ q3wk in phase II and III studies. Of these, only two patients (0.4%) had grade 3 CPK increase. Episodes of rhabdomyolysis (grade 3 and grade 4) were reported in two patients treated with single-agent lurbinectedin, but not at the RD of 3.2 mg/m^2^ q3wk. Consistently, in our study, only two cases of CPK increased (both grade1), one case of muscular weakness (grade 1), and one case of limb pain (grade 1) were reported. No case of rhabdomyolysis was reported in our study. Since no new safety limitations were found, lurbinectedin’s toxicities in our study were manageable and acceptable, as evidenced by the majority of the adverse reactions being relieved by appropriate therapeutic management. The incidences of treatment-related dose delay and reduction were also acceptable in our study. Moreover, the median cycles administered in our dose-expansion stage were similar to that in the basket trial (6 vs. 4). Notably, we did not include G-CSF primary prophylaxis, but it could be considered in future clinical practices, as G-CSF support could promote better tolerability^16^.

The efficacy of lurbinectedin was evaluated in both dose-escalation and dose-expansion stages, but mainly in the latter category, i.e., in the Chinese SCLC population, as second-line therapy. Regarding the efficacy evaluation in the dose-escalation stage, it was noted that two sarcoma patients benefited from lurbinectedin administration at 3.2 mg/m^2^, i.e., one soft tissue sarcoma patient had a confirmed PR as the best response, and another one with renal leiomyosarcoma displayed long-term stable disease. These two encouraging results were different from a previous phase II study results (NCT02448537), in which 42 metastatic soft tissue sarcoma patients (mostly leiomyosarcoma) were enrolled; however, 12 of them after treatment with lurbinectedin 3.2 mg/m^2^ did not respond. This might become a reminder of the indications of lurbinectedin development in the future. In the dose-expansion stage, all 22 enrolled patients showed a relapse after first-line platinum-based chemotherapy and were treated with the same dosage (i.e., 3.2 mg/m^2^, 1 h IV, q3wk) as the phase II basket trial. At the cutoff date, the result showed encouraging responses with an ORR of 45.5% (10/22, 95% CI 26.9–65.3) based on both IRC and investigator assessments, which was similar to the phase II basket trial result (35.2% by the investigator; 30.5% by IRC), and higher than the ORR (16.9%)^17^ reported with topotecan which was the only evidence-based standard of care in SCLC second-line therapy before the FDA’s approval of lurbinectedin. Notably, based on multiple small sample studies, the estimated ORR of topotecan in Chinese SCLC patients as second-line therapy ranged from 5%–30%. Other efficacy endpoint results in our study, e.g., mDOR, DCR, mPFS, and mOS, also presented similar or superior trends compared to the basket trial results (IRC-assessed) and the historical data of topotecan^17^: 4.2 months vs. 5.1 months vs. 4.2 months, 90.9% vs. 61.9% vs. 61.5%, 5.6 months vs. 3.5 months vs. 3.5 months, and 11.0 months vs. 9.3 months vs. 7.8 months, although some of our results were immature at cutoff.

For SCLC patients having a first-line therapy relapse, CTFI is considered the strongest predictor of outcome, as patients with the sensitive disease (CTFI ≥ 90 days) have a tumor response rate of 25% to additional chemotherapy, whereas patients with the resistant disease (CTFI < 90 days) exhibit tumor response rates < 10%^18^. The CTFI subgroup analysis in our study demonstrated that the lurbinectedin response in SCLC patients with the sensitive disease (CTFI ≥ 90 days), was better than that in the resistant population (CTFI < 90 days): 50% vs. 37.5%, by IRC and investigator assessments. This finding was consistent with (or even superior to) the basket trial results (sensitive vs. resistant: 43.3% vs. 13.3%, by IRC and 45.0% vs. 22.2%, by the investigator). Furthermore, our survival results (mOS, sensitive vs. resistant: 11.0 months vs. 9.2 months) were similar to the basket trial results (mOS, sensitive vs. resistant: 11.9 months vs. 5.0 months).

Immunotherapy in SCLC has considerably improved, as evidenced by the FDA’s approval for atezolizumab (2019)^19^ or durvalumab (2020)^20^ in combination with chemotherapy for the first-line therapy of extensive-stage SCLC^12^. The SCLC patients’ percentage who had prior immunotherapy (e.g., PD-1/PD-L1 inhibitors) in our study (dose-expansion stage) was much higher than the basket trial result (SCLC cohort): 63.6% (14/22) vs. 7.6% (8/105), which could be attributed to the different timings of both the studies. In patients who had prior immunotherapy, 35.7% (5/14) patients in our study and 62.5% (5/8) patients in the basket trial had responses (PRs, IRC assessed). Additionally, these results might indicate greater lurbinectedin’s efficacy in patients who had prior immunotherapy but should be interpreted with caution due to the limited sample size in both studies^4^.

Regarding the PK profile, lurbinectedin’s exposure in plasma increased with increased dosage, and the plasma concentration reached C_max_ at the end of infusion and decreased rapidly after the infusion. With an administration interval of 21 days, the plasma concentration before the second administration was below the Lower Limit of Quantification, and no drug accumulation was observed after two administration cycles. Compared with the basket trial results, the PK profile in Chinese patients was similar to that of Caucasian patients. Due to the small sample size, no exploration was conducted in our study to evaluate the relationships between lurbinectedin exposure and clinical safety and efficacy in Chinese SCLC patients. However, the exposure–response (E–R) analyses have been done by Pharma Mar to determine the correlation between lurbinectedin exposure and safety endpoints from phase I to III studies (n = 692) and also with efficacy endpoints from study B-005 (n = 99), and the results supported a favorable benefit-risk profile for lurbinectedin 3.2 mg/m^2^ q3wk^21^.

Since this was a bridging study with a small sample size, the potential bias cannot be excluded. However, as per the ICH guidance “E5 Ethnic Factors in the Acceptability of Foreign Clinical Data”, this study was designed by minimizing the duplication of clinical studies and supplying medicines expeditiously to patients for their benefit. In this regard, this study might have concluded at this stage. However, further evaluations of lurbinectedin in a broader population are warranted for greater validation.

## Conclusions

Our study met its primary efficacy endpoint, i.e., IRC-assessed ORR in the dose-expansion stage, which was higher than the phase II basket trial result of the SCLC cohort and much superior to the topotecan’s available data. The survival benefits of first relapsed Chinese SCLC patients from lurbinectedin treatment (3.2 mg/m^2^, 1 h IV, q3wk) were also observed, albeit some data were immature at cutoff. Together with a manageable and acceptable safety profile, our encouraging results can pave the way for lurbinectedin’s application as second-line therapy in Chinese adult patients with SCLC.

### Supplementary Information

Supplementary Information 1.

Supplementary Information 2.

## Data Availability

The datasets used and/or analyzed during the current study are available from the corresponding author on reasonable request.

## Abbreviations

SCLC: Small cell lung cancer
FDA: Food and Drug Administration
RD: Recommended dose
ICD: Immunogenic cell death
TAMs: Tumor-associated macrophages
NCCN: National Comprehensive Cancer Network
ORR: Overall response rate
mDOR: Median duration of response
mPFS: Median progression-free survival
mOS: Median overall survival
DLT: Dose-limiting toxicity
PK: Pharmacokinetics
G-CSF: Granulocyte colony-stimulating factor
RECIST: Response evaluation criteria in solid tumors
CT: Computed tomography
MRI: Magnetic resonance imaging
IRC: Independent review committee
PR: Partial response
CTFI: Chemotherapy-free interval
TEAEs: Treatment-emergent adverse events
SAEs: Severe adverse events
