# Supplementary material for: A pivotal bridging study of lurbinectedin as second-line therapy in Chinese patients with small cell lung cancer
Source: Sci Rep. 2024 Feb 13;14:3598. doi: 10.1038/s41598-024-54223-5 (PMC10864288; doi:10.1038/s41598-024-54223-5)
Supplement: Supplementary file 1 — Supplementary Information 1. [file 41598_2024_54223_MOESM1_ESM.docx]

**Main eligibility criteria**

The patients enrolled in the dose-escalation stage had solid tumors that did not respond to, refused, or could not tolerate standard treatment, and who had received ≤3 prior chemotherapy lines. While the dose-expansion stage patients had relapsed SCLC after first-line platinum-based chemotherapy. For both stages, the enrolled patients were ≥18 years, with measurable disease as per the Response Criteria in Solid Tumors (RECIST; version 1.1) and documented progression before the enrolment; had an Eastern Cooperative Oncology Group (ECOG) performance status <2; adequate bone marrow (evaluated by laboratory tests for the absolute neutrophil count, platelet count, and hemoglobin), kidney (evaluated by serum creatinine or creatinine clearance), and liver (evaluated by bilirubin, albumin, and aminotransferases, etc.) functions. Only patients with ≤grade 1 toxicities from any previous therapies were included, except for alopecia and peripheral sensory neuropathy (both grade 2) cases, which were also allowed. The minimum interval between any previous treatment and study commencement was 3, 4, and 2 weeks for chemotherapy, immunotherapy, or radiotherapy, and any investigational therapy or palliative radiotherapy, respectively. Evidence of non-childbearing status had to be obtained for women of childbearing potential (WOCBP). WOCBP should have used adequate contraception during the study and for at least 6 months after treatment discontinuation. Fertile male patients with WOCBP partners should use condoms during treatment and for 4 months following the last investigational medicinal product dose.

Patients were excluded if they had: previously received lurbinectedin or trabectedin; known central nervous system (CNS) involvement (screening of baseline CNS metastases was mandatory); concomitant unstable or serious medical condition within the past year (history or presence of unstable angina, myocardial infarction, congestive heart failure, arrhythmia, and active infection, etc.); the impending need for systemic antitumor therapy; those who received cytochrome P450 3A4 enzyme (CYP3A4) strong inducer or an inhibitor within 2 weeks (or 5 half-lives, whichever was longer), G-CSF treatment or blood transfusion within 2 weeks, or erythropoietin within 3 weeks before the first dose, and those who received or were unable to comply with the study protocol.
