# Supplementary material for: A pivotal bridging study of lurbinectedin as second-line therapy in Chinese patients with small cell lung cancer
Source: Sci Rep. 2024 Feb 13;14:3598. doi: 10.1038/s41598-024-54223-5 (PMC10864288; doi:10.1038/s41598-024-54223-5)
Supplement: Supplementary file 2 — Supplementary Information 2. [file 41598_2024_54223_MOESM2_ESM.docx]

**Supplementary Table S1** Brief Summary of Adverse Events in Dose-escalation Stage and Dose-expansion Stage

|  | Dose-escalation | | Dose-expansion |
| --- | --- | --- | --- |
|  | 2.5mg/m^2^ | 3.2mg/m^2^ | 3.2mg/m^2^ |
| Patients with: | n (%) | n (%) | n (%) |
| At least one TEAE regardless of relationship | 3 (100%) | 7 (100%) | 22 (100%) |
| Any treatment-related (or with unknown relationship) AE | 3 (100%) | 7 (100%) | 22 (100%) |
| Any grade ≥3 TEAE | 2 (66.7%) | 7 (100%) | 19 (86.4%) |
| Any grade ≥3 treatment-related (or with unknown relationship) AE | 2 (66.7%) | 7 (100%) | 19 (86.4%) |
| Any treatment-emergent SAE | 1 (33.3%) | 1 (14.3%) | 11 (50%) |
| Any treatment-related (or with unknown relationship) SAE | 1 (33.3%) | 1 (14.3%) | 11 (50%) |
| Any grade ≥3 treatment-emergent SAE | 1 (33.3%) | 1 (14.3%) | 11 (50%) |
| Any grade ≥3 treatment-related (or with unknown relationship) SAE | 1 (33.3%) | 1 (14.3%) | 11 (50%) |
| TEAEs leading to death | 0 | 0 | 0 |
| TEAEs leading to treatment discontinuation | 0 | 2 (28.6%) | 1 (4.5%) |

TEAE, Treatment-emergent Adverse Events; AE, Adverse Events; SAE, Severe Adverse Events.

**Supplementary Table S2**  PK Parameters after 2.5mg/m^2^ and 3.2mg/m^2^ Dosage Infusion

|  | 2.5mg/m^2^ | | 3.2mg/m^2^ | |
| --- | --- | --- | --- | --- |
|  | Cycle 1 (n=3) | Cycle 2 (n=3) | Cycle 1 (n=21) | Cycle 2 (n=15) |
| C_max_ (ng/mL) | 107.534±14.185 | 111.12±26.439 | 174.494±189.179 | 117.685±53.027 |
| AUC_0-∞_ (h*ng/mL) | 311.474±112.480 | 517.760±267.840 | 552.495±281.457 | 834.310±690.350 |
| V_z_ (mL) | 1040516.997±317313.083 | 379246.471±143435.260 | 1031063.961±576486.886 | 316465.365±158476.151 |
| CL (mL/h) | 14730.778±5419.721 | 9559.551±4176.787 | 12311.041±5038.516 | 12712.641±9466.587 |
| t_1/2_ (h) | 51.578±15.026 | 28.241±2.614 | 58.708±19.719 | 31.575±14.684 |

PK, Pharmacokinectics; Cmax, Peak Concentration; AUC, Area Under Curve; V_z_, Apparent Volume of Distribution; CL, Clearance; t_1/2_, Half-time. Data are presented as mean ± standard deviation.


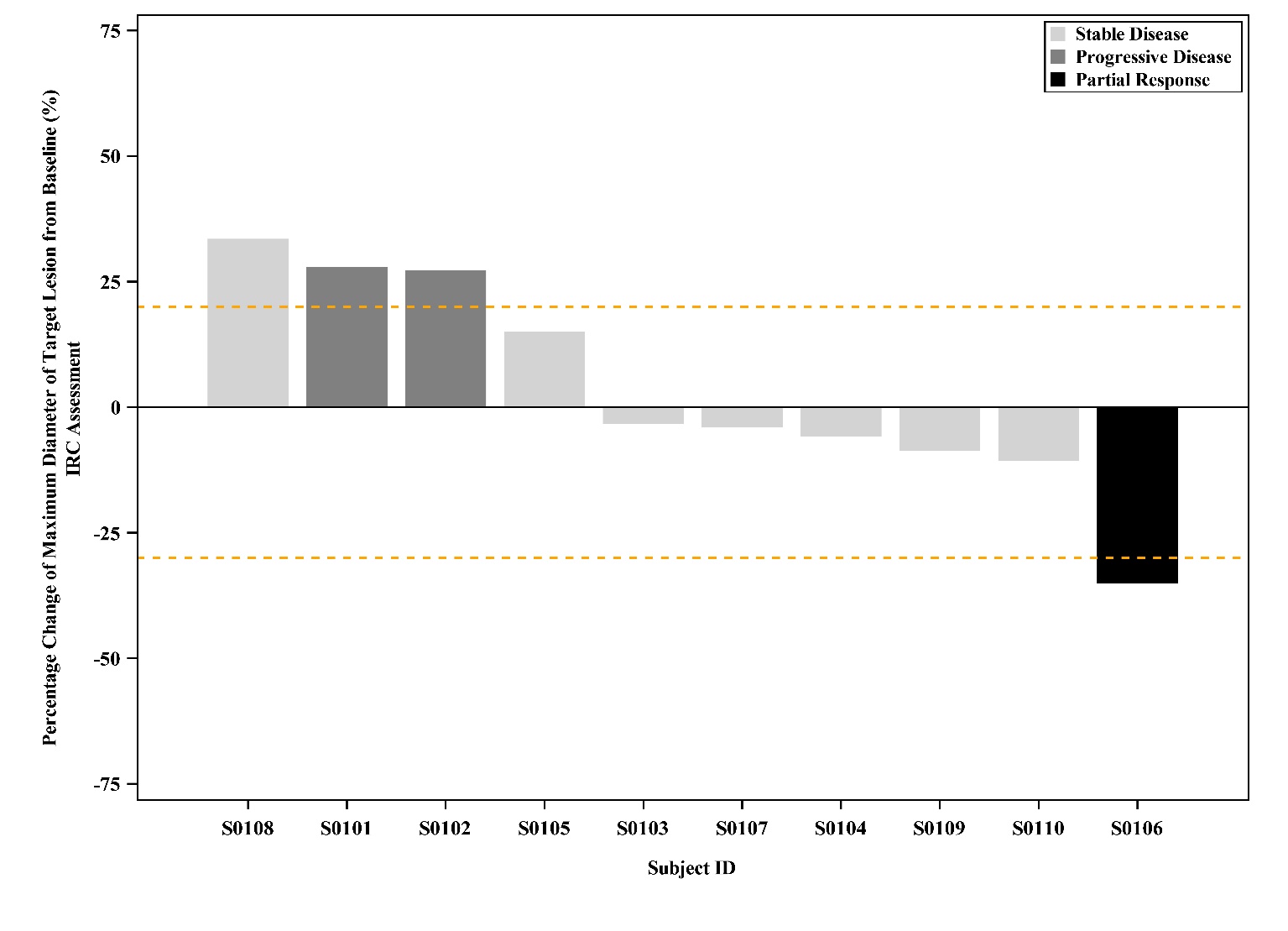


**Supplementary Fig. S1** Waterfall plot showing maximum variation of target lesions size (n=10) and the confirmed best response in the dose-escalation stage (IRC assessment). IRC, Independent Review Committee


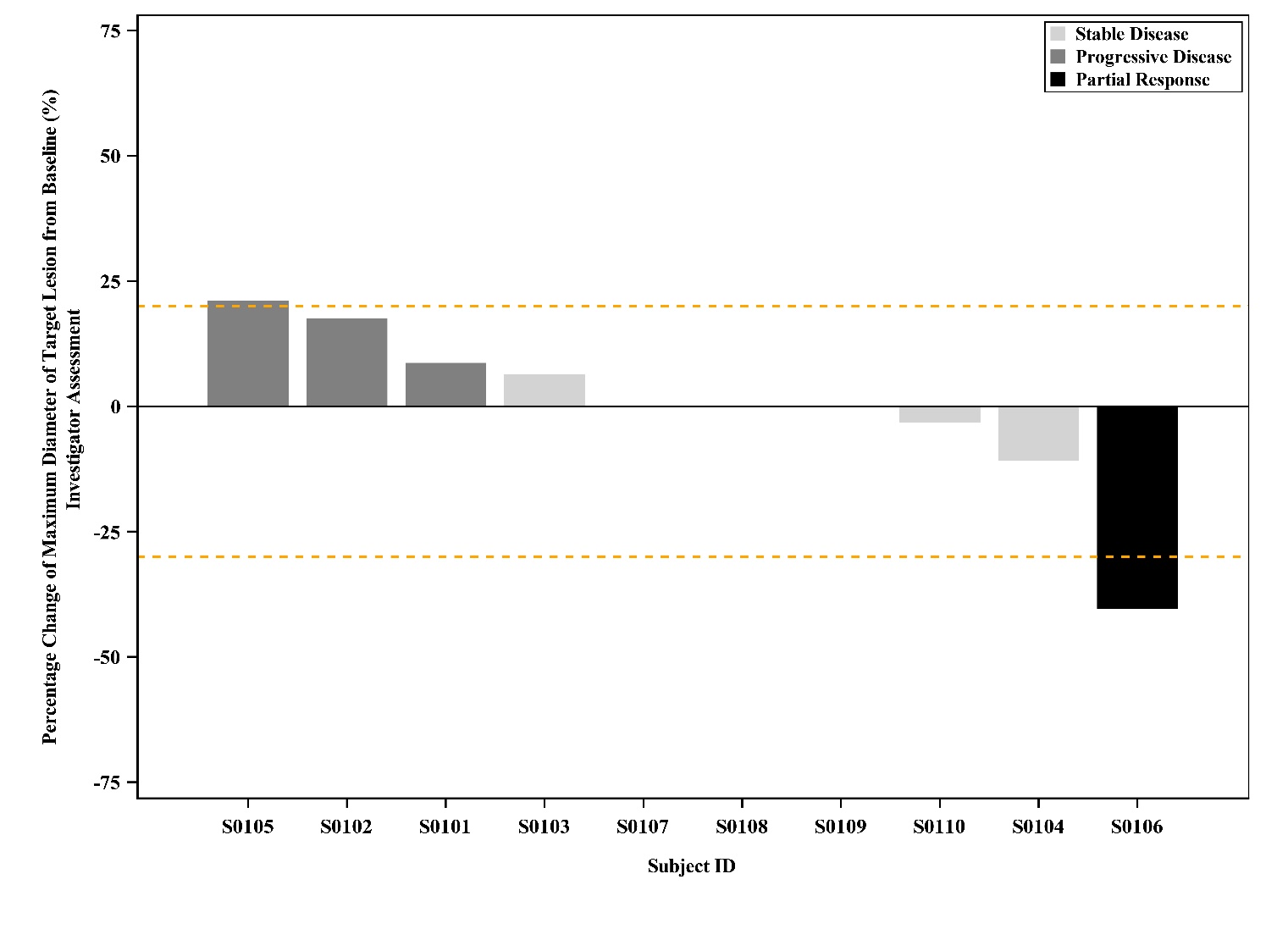


**Supplementary Fig. S2** Waterfall plot showing maximum variation of target lesions size (n=10) and the confirmed best response in the dose-escalation stage (Investigator assessment). S0107, S0108, S0109: the variation of target lesion size = 0


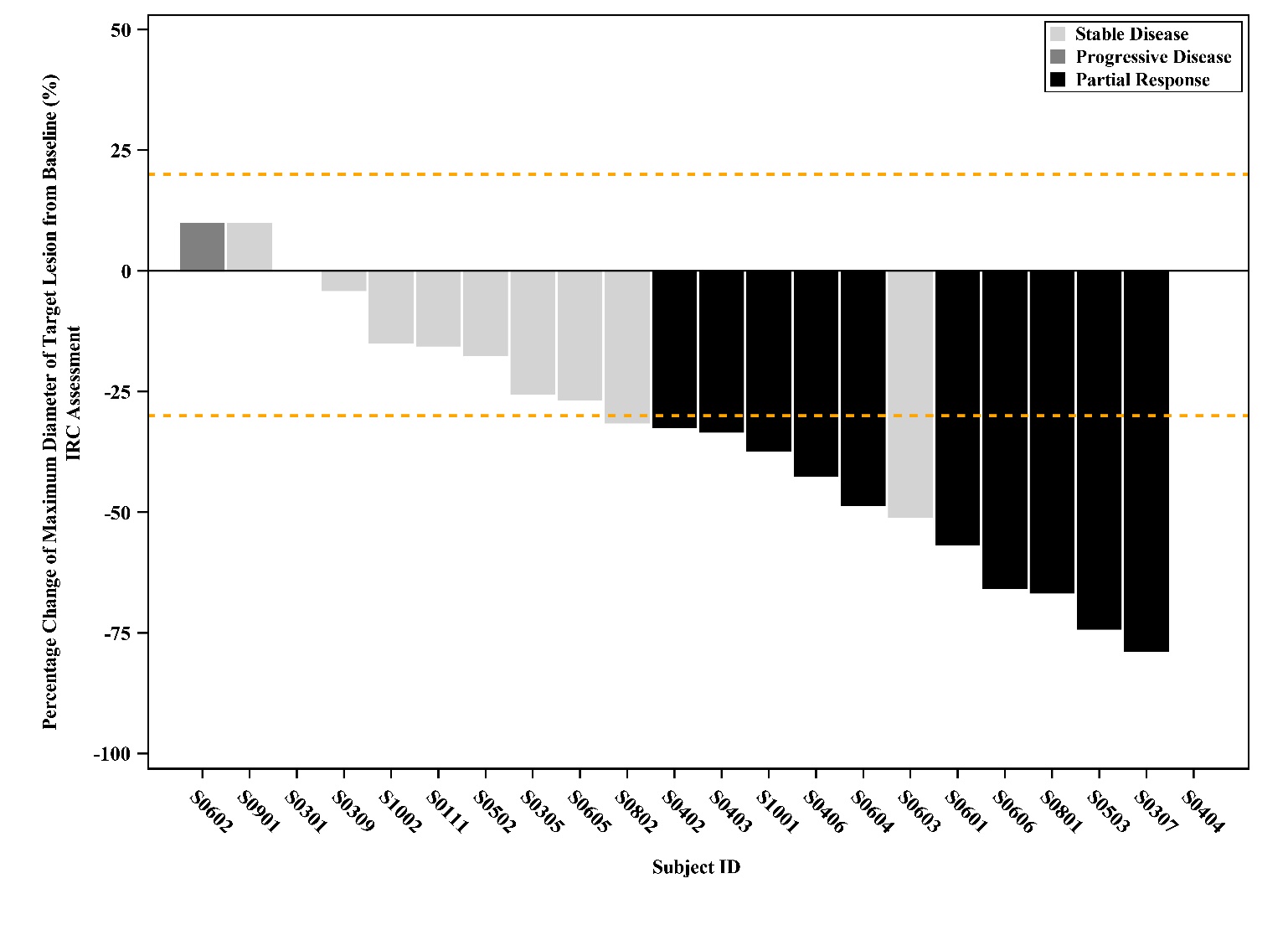


**Supplementary Fig. S3** Waterfall plot showing maximum variation of target lesions size (n=22) and the confirmed best response in the dose-expansion stage (IRC assessment). S0301: the variation of target lesion size = 0; S0404: not evaluable; IRC, Independent Review Committee.


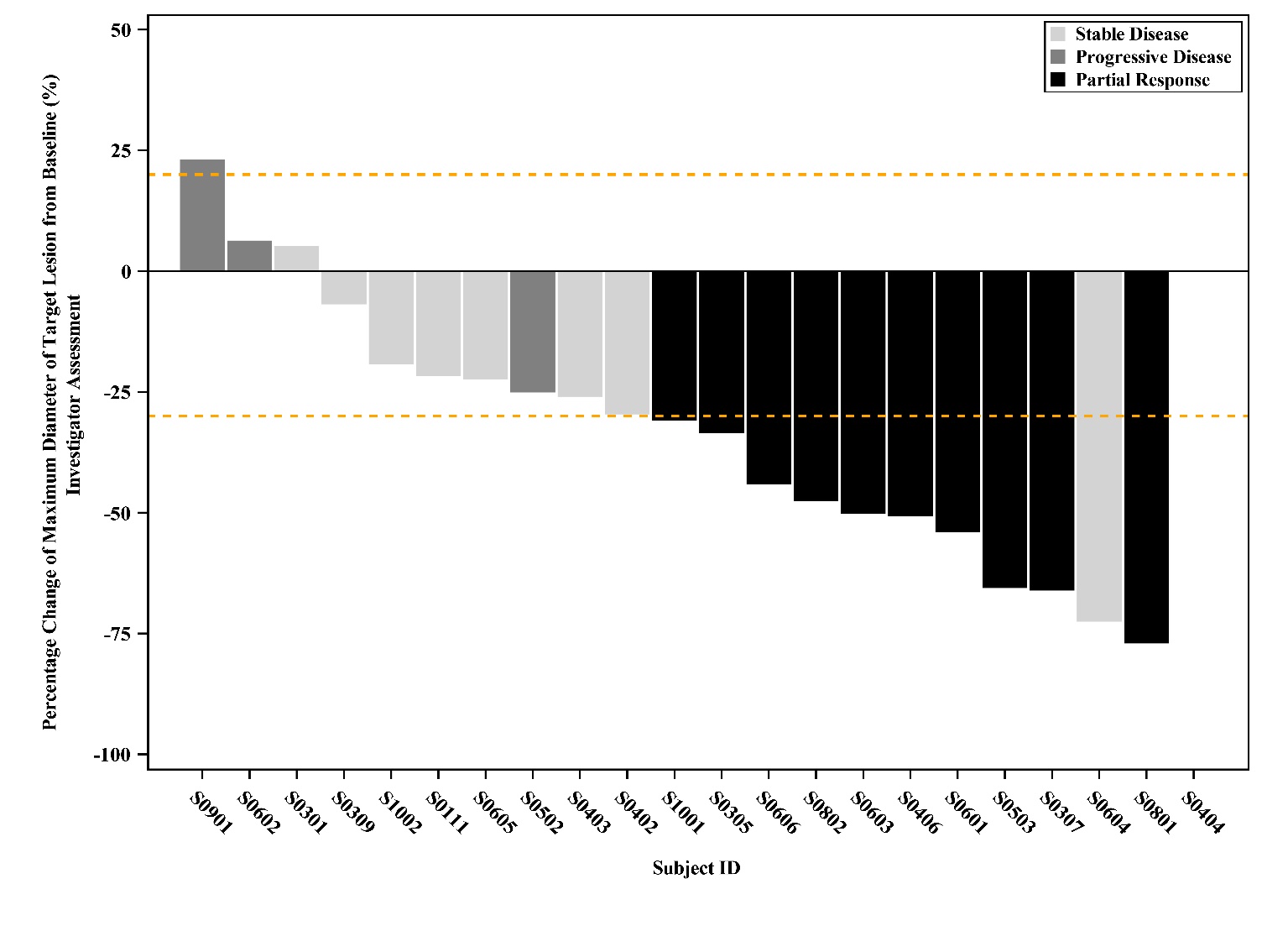


**Supplementary Fig. S4** Waterfall plot showing maximum variation of target lesions size (n=22) and the confirmed best response in the dose-expansion stage (Investigator assessment). S0404: not evaluable


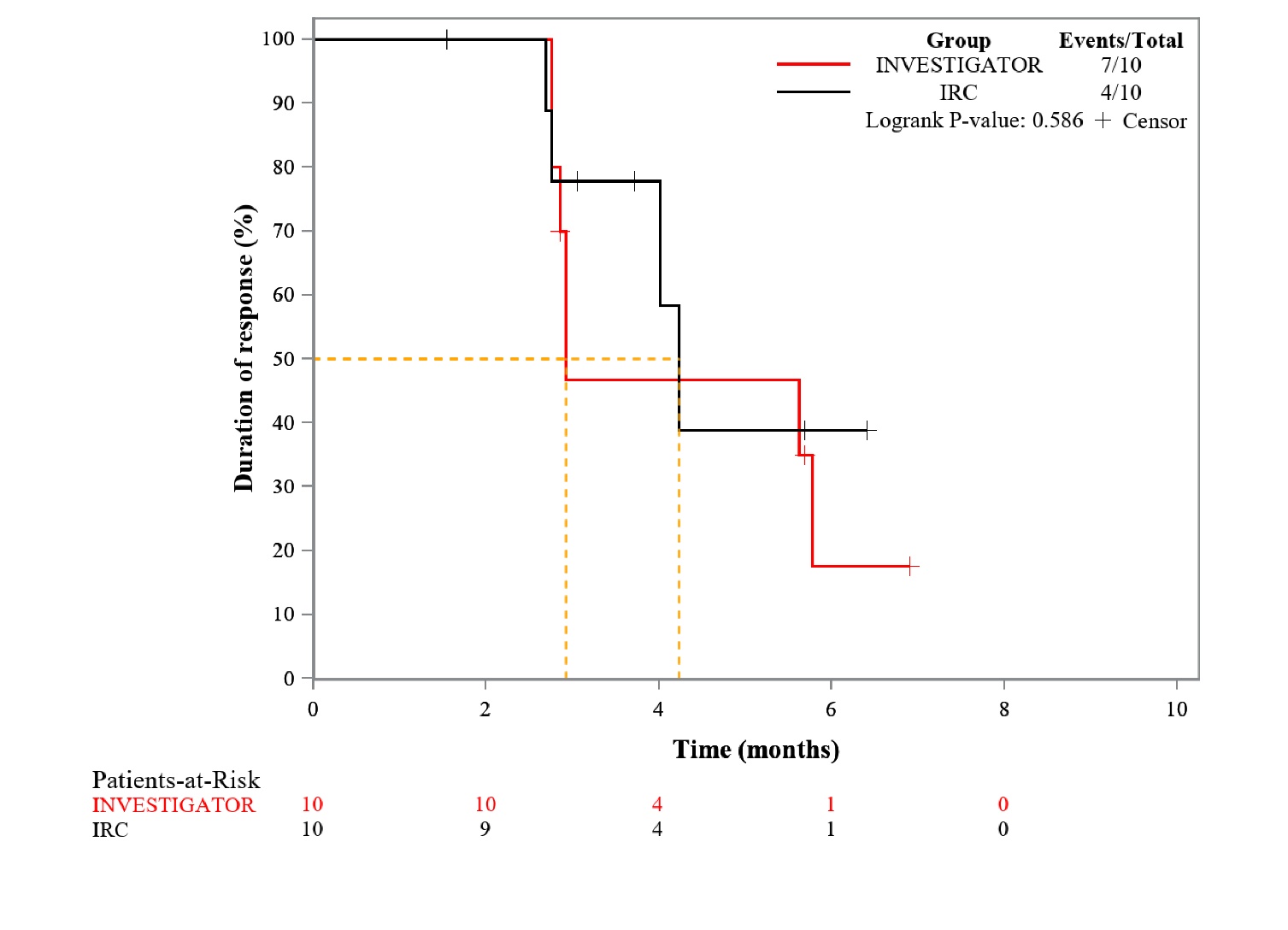


**Supplementary Fig. S5** Kaplan-Meier plot of DOR by the Investigator assessment and IRC assessment in the dose-expansion stage. DOR, Duration of Response; IRC, Independent Review Committee


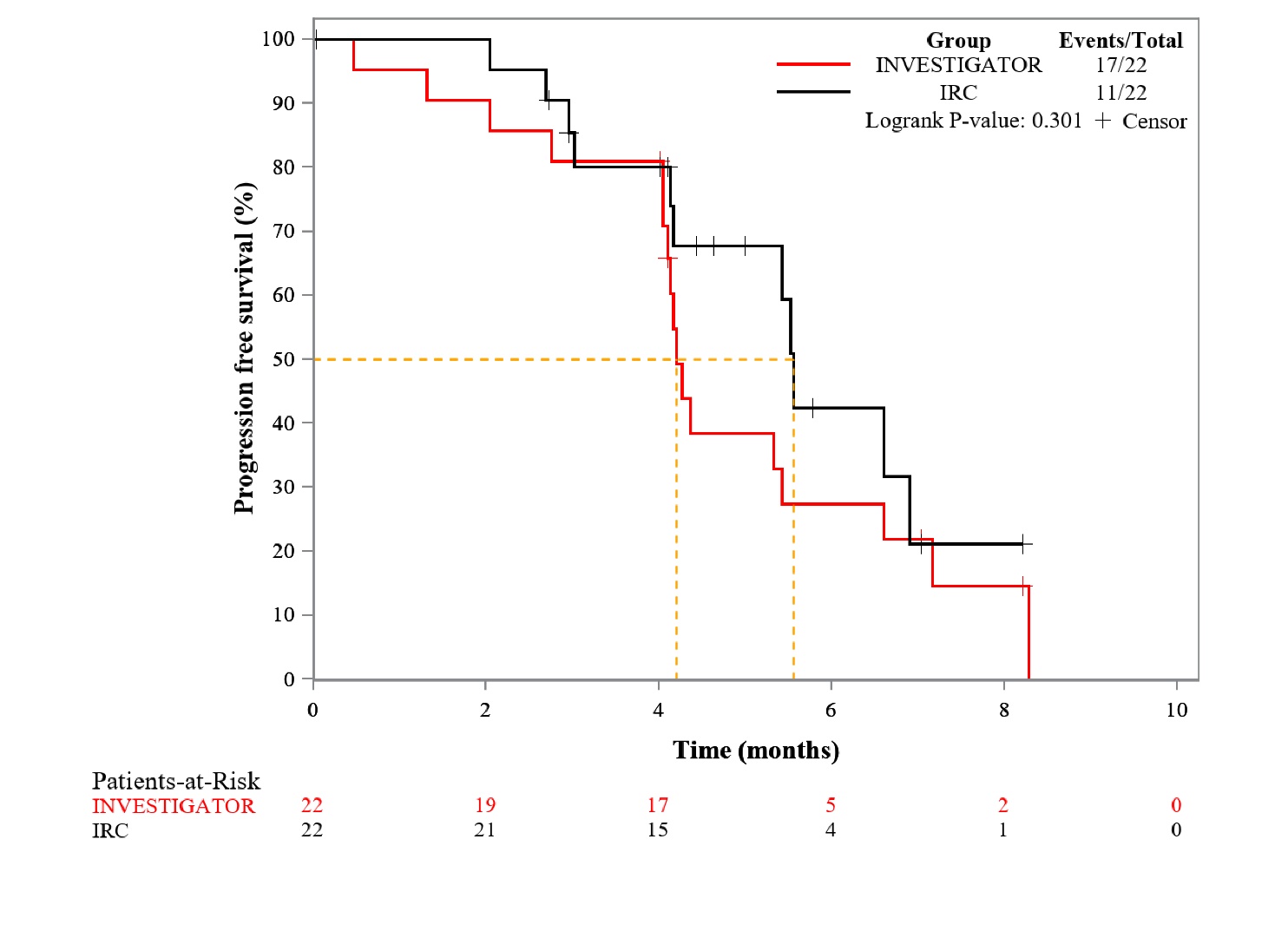


**Supplementary Fig. S6**  Kaplan-Meier plot of PFS by Investigator assessment and IRC assessment in the dose-expansion stage. PFS, Progression-free Survival; IRC, Independent Review Committee


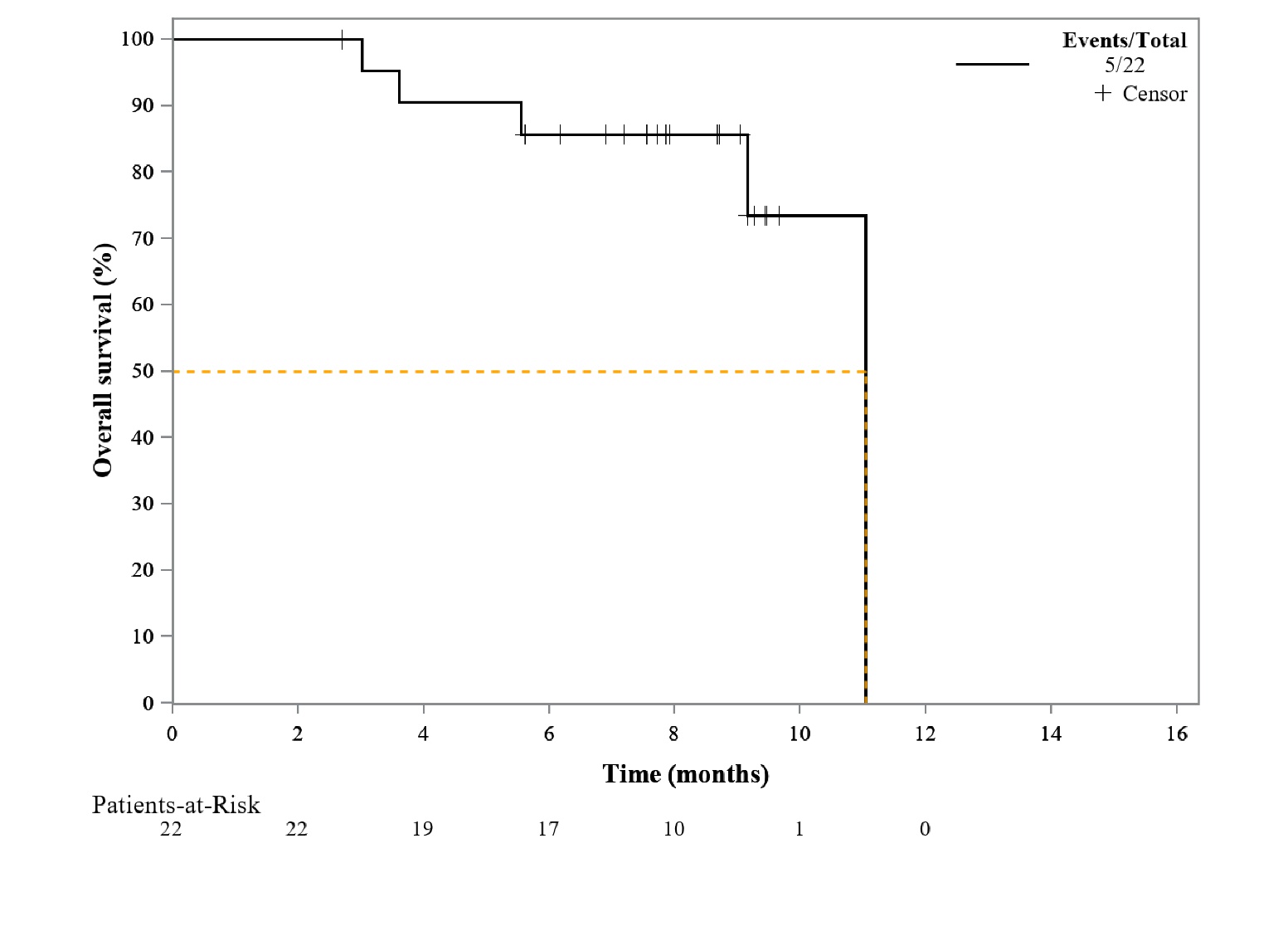


**Supplementary Fig. S7** Kaplan-Meier plot of overall survival in the dose-expansion stage (n=22 treated patients)


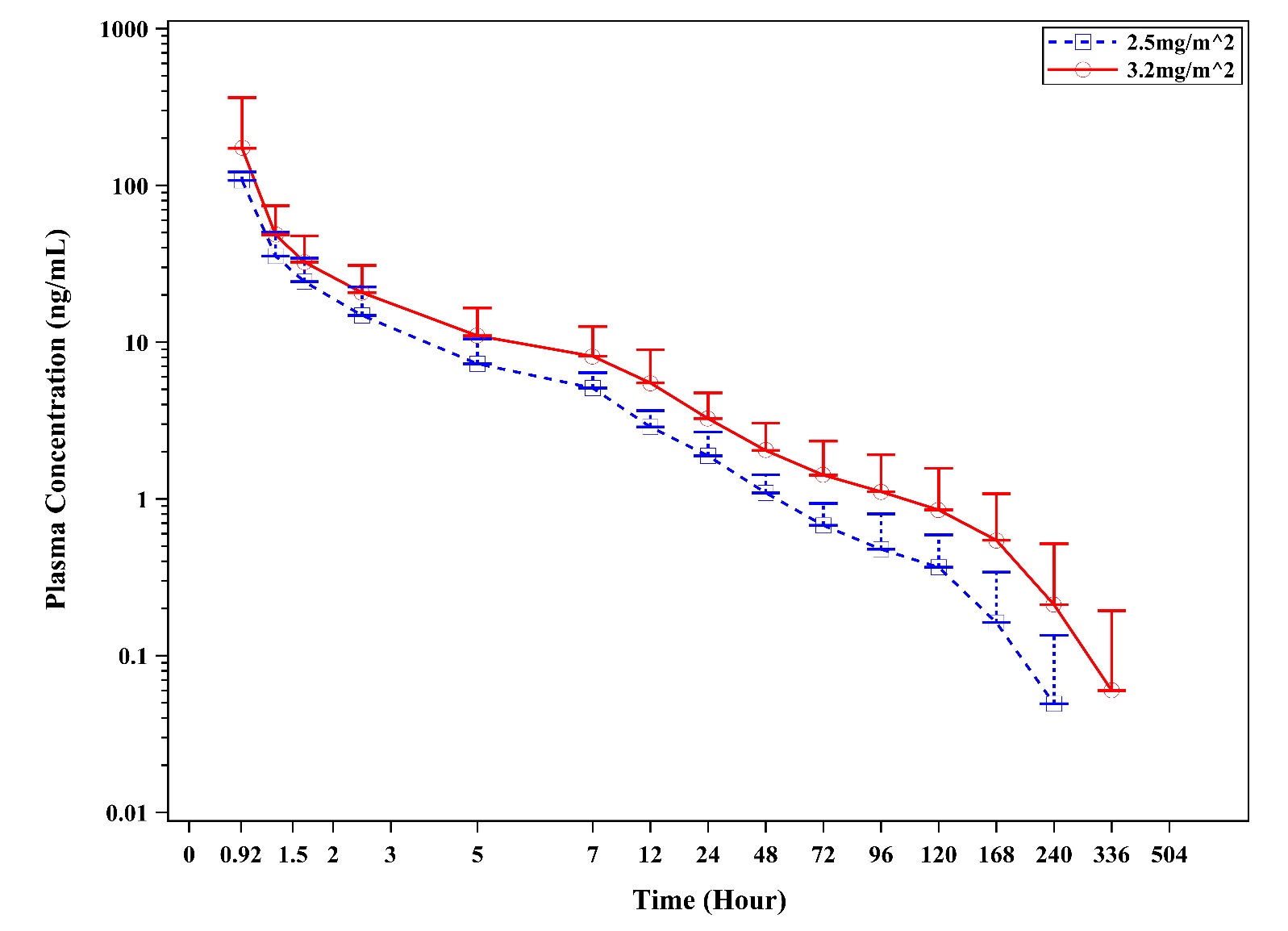


**Supplementary Fig. S8** Plasma drug concentration-time curve of Cycle 1

**
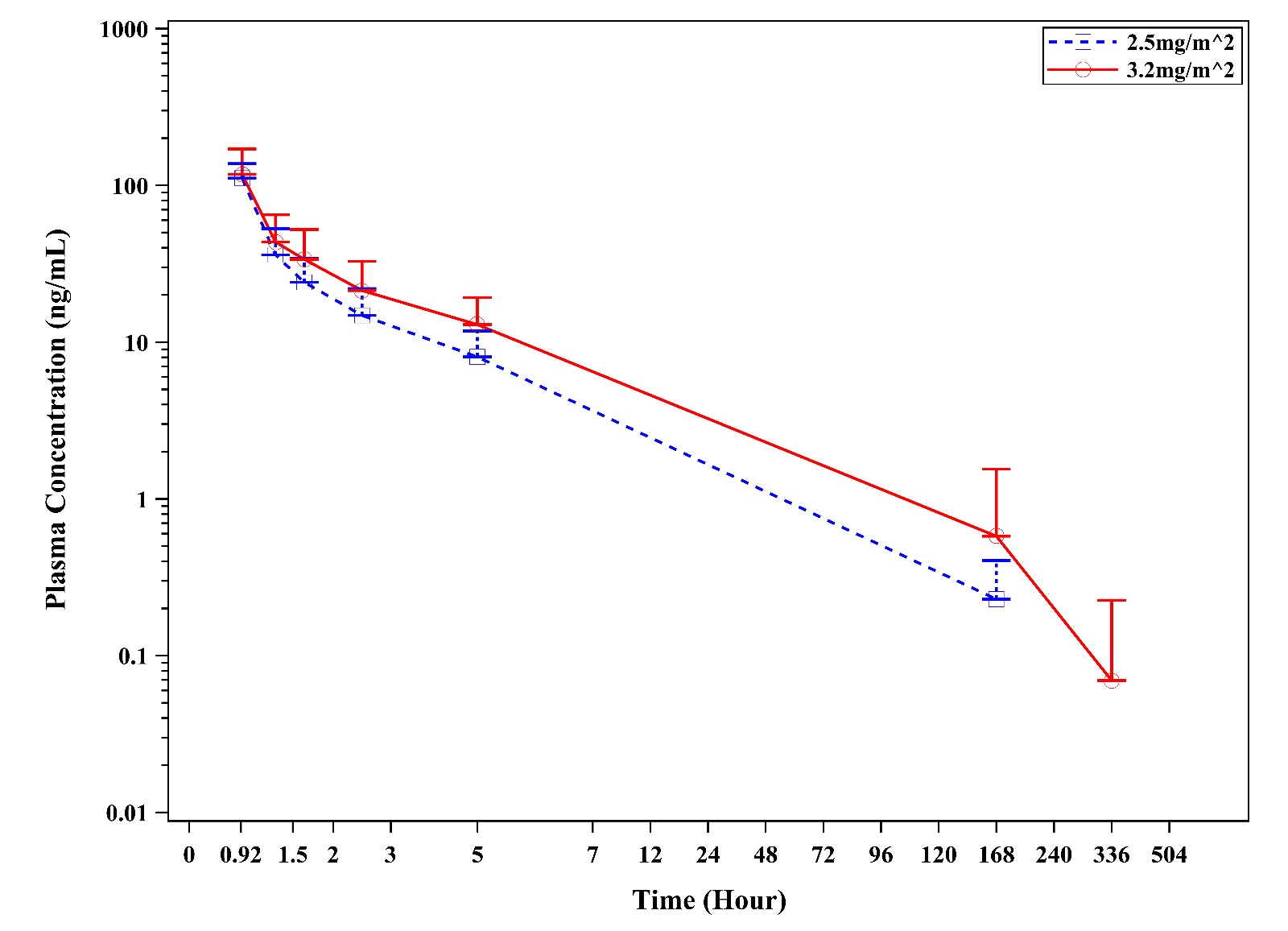
**

**Supplementary Fig. S9** Plasma drug concentration-time curve of Cycle 2
